# Structural Basis of Activity of and Resistance to HIV Integrase Inhibitors

**DOI:** 10.1063/4.0001061

**Published:** 2025-10-27

**Authors:** Dmitry Lyumkis

**Affiliations:** Salk Institute for Biological Studies, La Jolla, CA, USA

## Abstract

The Human Immunodeficiency Virus Type 1 (HIV) currently infects ∼40 million people worldwide. In the absence of a cure, antiretroviral therapy represents the primary treatment option, because it slows disease progression and reduces new infections. Integrase (IN) Strand Transfer Inhibitors (INSTIs) are a class of antiretroviral therapeutics that block integration of viral DNA into host chromosomes. This process is mediated by the viral IN enzyme, which assembles into oligomeric nucleoprotein complexes on the ends of viral DNA, termed “intasomes”. INSTIs selectively target intasomes and represent first-line therapies in the clinic. However, the emergence of IN variants resistant to INSTIs is becoming a greater clinical problem. We are using interdisciplinary approaches that include structural biology via cryogenic electron microscopy, biochemistry, cellular virology, and computation to provide a mechanistic understanding of both how and why select drug resistant mutations arise in response to leading clinically used drugs, identify and analyze novel pathways of drug resistance, and devise strategies to predict how resistant mutations emerge. We are also working with chemists who are developing more potent therapeutics that aim to be effective against drug resistant viruses. Our work aims to improve our understanding of an important class of drugs used to treat people living with HIV, identify mechanisms, pathways, and patterns of clinically relevant resistance to INSTIs, and provide specific guidelines for their rational improvement, under a broader umbrella of “personalized medicine”.

